# The Relationship between the 24 h Blood Pressure Variability and Carotid Intima-Media Thickness: A Compared Study

**DOI:** 10.1155/2014/303159

**Published:** 2014-02-11

**Authors:** Huahua Xiong, Dan Wu, Xiaohong Tian, Wan-Hua Lin, Chunyue Li, Heye Zhang, Yunpeng Cai, Yuan-Ting Zhang

**Affiliations:** ^1^Department of Ultrasound, The Second Peoples' Hospital of Shenzhen, Shenzhen 518029, China; ^2^Institute of Biomedical and Health Engineering, Shenzhen Institutes of Advanced Technology, Shenzhen 518055, China; ^3^Key Lab for Health Informatics, Chinese Academy of Sciences, Shenzhen 518055, China; ^4^Cardiac Electrocardiogram Room, The Second Peoples' Hospital of Shenzhen, Shenzhen 518029, China; ^5^Joint Research Center for Biomedical Engineering, The Chinese University of Hong Kong, Shatin, New Territories, Hong Kong

## Abstract

Large blood pressure variability (BPV) will not only harm the target organ but also increase the possibility of the cardiovascular events. Since the damage of vascular system always leads to the alteration of the carotid wall, the structure and function of the carotid artery have been extensively examined in previous studies. In this work we conduct a study (60 subjects, aged 33–79) to evaluate the relationship between BPV and carotid intima-media thickness (IMT) in Shenzhen, which is one large city in the southern area of China. In our study, the blood pressure (BP) was collected using the 24 h ambulatory BP monitoring, and the BPV was evaluated using standard deviation (SD), coefficient of variation (CV), and average real variability (ARV) during 24 h, daytime and nighttime. All the IMT measurements are collected by ultrasound. The results show that both the daytime, and 24 h systolic BPV evaluated by three indices are positively associated with IMT. Among them, daytime systolic BPV evaluated with ARV is the best variable to represent the increasing of carotid IMT. In addition, after adjusting by age, sex, smoking, hypertension, and mean BP and PP values, 24 h diastolic BPV evaluated with SD also presents the favorable performance.

## 1. Introduction

A large number of studies have suggested that hypertension can be the potential cause of cardiovascular disease (CVD) [1–3]. Therefore, the blood pressure (BP) level becomes one risk factor that has been frequently examined in the previous studies [4, 5]. Recently, blood pressure variability (BPV) has been proved to be promising in providing potential regulatory mechanisms of the cardiovascular system [6], and it can be simply classified as the short-term BPV and long-term BPV. Although the long-term BPV is strongly prognostic of cardiovascular morbidity and mortality [7], it is a very time-consuming process to collect the visit-to-visit BP measurements from the patients and calculate the BPV for each of them, and it may take more than several years to finish the whole study. However, for the short-term BPV analysis, we could obtain the extensive information on BP profile and identify the patterns of circadian BP variation using the ambulatory blood pressure measurement (ABPM) [8]. Therefore, researchers are looking for the BPV analysis on ABPM; for example, from the analysis of ABPM, 24 h mean BP, different indices of BPV, and day-to-night BP changes can be obtained. Indeed, some studies have found that the BPV calculated from ABPM data could be a valuable predictor for progression of subclinical organ damage and also a risk factor for cardiovascular mortality in the Japanese population [9, 10]. Because of high economical development speed in China, the CVD and stroke are more often found in the southern area of China, which is traditionally considered as low endemic area. Hence, it is worthy to conduct a study to examine the prognostic significance of ABPM and its BPV over the population in the southern area of China.

In order to evaluate the effect of BPV on the carotid artery alteration or the damage progress of organ, one straightforward way is to examine the correlation between the BPV and the structure of vascular vessel [11, 12]. For the change of vascular structure, B-mode ultrasound is one widely applied tool to assess the carotid artery imaging and measure the carotid intima-media thickness (IMT). The increased IMT can be used as one predictor of the atherosclerosis and it is also a predictor for the cardiovascular events associated with the incident stroke [13, 14].

Accordingly, it will be one meaningful topic to examine the effects of different indices of BPV on the carotid artery alteration especially in the vascular structures over the population of the southern area in China. Stabouli et al. have reported that the carotid IMT was positively correlated to systolic ABPM value, but not the diastolic value [15]. It indicates that the systolic blood pressure (SBP) has a strong relation to the vascular lesions. In the study of García-García et al. [16], they found that systolic blood pressure variability (SBPV) had positive relationship with pulse wave velocity, and ambulatory arterial stiffness index in hypertensive patients. Zakopoulos et al. evaluated the time rate of BP and the extent of common carotid artery IMT in the normotensive and hypertensive subjects [17]. Although several studies have suggested that the increased BPV is associated with an increase in subsequent cardiovascular events, there are other studies where the cited association was not found [18, 19]. An explanation for these apparently contradictory results may be the selection of the index used for quantifying variability.

Thus, the aim of this study is to find out the possible relationship between the 24 h ambulatory BPV and carotid IMT over the population of the southern area in China. Furthermore, IMT has been well recognized as one predictive factor of CVD, and the change of BPV will definitely affect the structure and function of the carotid artery. Hence, it is worthy to evaluate the correlation between the carotid IMT and the BPV and compare the prognostic significance of different indices of BPV.

## 2. Methods

### 2.1. Study Design and Population

The study was conducted in the Second Peoples' Hospital of Shenzhen, Guangdong Province, China. 60 individuals aged 33–79 years (53.3% male gender) were enrolled in this study. They fulfilled the following inclusion criteria: (1) no history or clinical evidence of diabetes mellitus (fasting serum glucose <7.0 mmol/L; nonfasting serum glucose <11.1 mmol/L); (2) both 24 h BP monitoring and carotid artery ultrasound measurement were performed; (3) the valid BP measurements within 24 h ≥90%. This study was approved by the Institutional Ethics Committee of the Second Peoples' Hospital of Shenzhen (China), and the informed consent was obtained from every subject.

### 2.2. Ambulatory Blood Pressure Measurement

All of the subjects underwent 24 h ABPM on a day of daily activity. A proper cuff was selected according to the size of subject's arm and placed on the nondominant arm. The subjects were asked to keep their arms still at the time of measurements. The ambulatory BP was recorded automatically using a commercial device (Mobil Graph 24 h ABP-Control). The daytime BP monitoring was from 7:00 to 22:59, measured automatically every 30 minutes, and during the nighttime, from 23:00 to 6:59, the BP was measured once an hour.

According to the recorded 24 h BP measurements, BPV was evaluated through the standard deviation (SD), coefficient of variation (CV), and average real variability (ARV) of the SBP and diastolic BP (DBP) during daytime, nighttime, and over 24 hours. For the short-term (with 24 h) BPV analysis, SD, CV, and ARV are the common indices of BPV in time domain. SD is calculated by the following formula

(1) $$\begin{array}{c} \text{SD}=\sqrt{\frac{1}{N-1}\overset{k=N}{\underset{k=1}{\sum }}{\left({\text{BP}}_{k}-{\text{BP}}_{\text{mean}}\right)}^{2}}, \end{array}$$

where *N* is the number of valid BP measurements. The average SD of BP can also be divided by the corresponding mean BP and multiplied by 100 to express a normalized measure of BPV as a CV; the formula is as follows

(2) $$\begin{array}{c} \text{CV}=\frac{\text{SD}}{\text{mean}}\times 100\%. \end{array}$$

ARV showed the average of the absolute differences between consecutive BP measurements, which was calculated as follows:

(3) $$\begin{array}{c} \text{ARV}=\frac{1}{N-1}\overset{N-1}{\underset{k=1}{\sum }}\left|{\text{BP}}_{k+1}-{\text{BP}}_{k}\right|, \end{array}$$

where *N* is also the number of valid BP measurements.

### 2.3. Carotid Artery Ultrasound Examination

The carotid artery ultrasound was examined using a high-resolution ultrasound Doppler system (iU22, Philips Ultrasound, Bothell, WA, USA), with a 7.5 MHz liner array transducer. During the examination, the subjects were supine in the bed, with the head turned 45° away from the examined side. The left and right common carotid arteries, carotid bulbs, and internal carotid arteries were scanned in three angles (lateral, anterior, and posterior). Thus, we can assess the mean IMT in each position from the three measurements in different angles. The specific places we measured in the carotid artery were defined as follows: the IMT at the common carotid artery was measured on the far wall of blood vessel, 10–20 mm proximal to the carotid bifurcation. The carotid bulb we measured was in the carotid bifurcation, and the IMT at the internal carotid artery was measured over a distance of 10–20 mm from the bifurcation. In our study, the correlation analysis will focus on the common carotid artery, and thus carotid IMT in this paper represents the IMT at the common carotid artery, which is an average of right and left IMT. Besides, the abnormal IMT is defined that the IMT at the common carotid artery is more than 1.0 mm.

### 2.4. Statistical Analysis

The correlation between the BPV and the IMT was analyzed using a two-tailed Pearson's test. When the correlation coefficient *r* was close to 1, it indicated that the BPV had highly positive correlation with IMT. On the contrary, when *r* was close to −1, the relativity about BPV and IMT was negative. *P* value < 0.05 was considered statistically significant. Furthermore, simple linear regression analysis was applied to detect the linear relationship between the BPV indices and IMT. Besides, multiple linear regression analysis was performed to estimate the relation between different indices of BPV and IMT. We defined the carotid IMT as the dependent factor and the BPV estimated with SD, CV, and ARV as the independent factors, respectively. Finally, we carried out a multiple linear regression analysis using the backward selection method to verify which BPV index was independent of the baseline characteristics, mean BP, and pulse pressure (PP) values. All of the analyses were performed using SPSS 15.0 statistical package (SPSS Inc., Chicago, Illinois, USA).

## 3. Results

Among all of the participants, we excluded the cases that had the incomplete or invalid measurements. Finally, a total of 60 patients aged 33–79 (male 53.3%) were successfully obtained in the study. Of those, 26 subjects had the normal carotid IMT, and 34 subjects had a carotid IMT more than 1.0 mm, which is defined as the abnormal IMT.  Table 1 summarized the clinical characteristics of all the subjects and two subgroups: the subjects with the normal IMT and the subjects with the abnormal IMT. The data of clinical characteristics were expressed as means ± SDs or percentages. In this table, mean SBP, DBP, and PP values in different periods of time, BP decreasing percent from day to night, IMTs at different carotid arteries, the plaque, and smoking status were reported. No significant differences were documented between the normal IMT group and abnormal IMT group regarding the BP values. However, for the baseline characteristics, the subjects in the abnormal IMT group were significantly older than the subjects in the normal IMT group (*P* < 0.001). Besides, in the abnormal IMT group, 67.6% of subjects had the plaques, which is higher than that in the normal IMT group (*P* < 0.001). Moreover, the abnormal IMT group had a significantly greater IMT both at bulb and internal carotid artery than the normal IMT group (*P* < 0.001), and most of them tended to suffer from the prevalence of the atherosclerotic plaques (*P* < 0.001).

We evaluated the BPV using SD, CV, and ARV, and the average BPV values quantified with three indices in 24 h were reported in Table 2. We compared the correlations of these BPV values in each group of the two using Pearson's test. No significant differences were found among the three indices of BPV; they had strongly positive correlation (*P* < 0.01). Moreover, we found that all of the SBPV values were greater than those of DBPV when evaluated using SD and ARV. In contrast, the DBPV values were found to be greater than SBPV when using CV as a measure.

**Figure 1:**
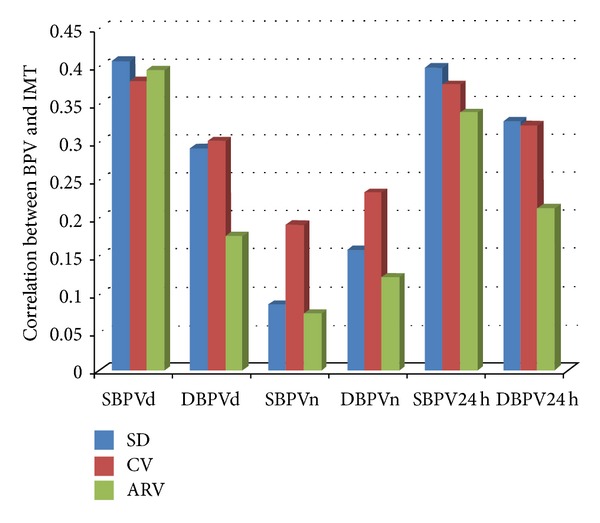
The correlation of the blood pressure variability and carotid intima-media thickness in all subjects. BPV, blood pressure variability; IMT, intima-media thickness; CV, coefficient of variation; ARV, average real variability; SBPV, systolic blood pressure variability; DBPV, diastolic blood pressure variability; SBPVd, daytime SBPV; DBPVd, daytime DBPV; SBPVn, nighttime SBPV; DBPVn, nighttime DBPV; SBPV24 h, 24 h SBPV; DBPV24 h, 24 h DBPV.

Table 3 depicted the correlation coefficients between different indices of BPV and carotid IMT in all subjects. As the results showed, for all the subjects, the SD, CV, and ARV of daytime SBP showed a positive correlation with IMT (*r* = 0.408, *P* = 0.001; *r* = 0.381, *P* = 0.003; *r* = 0.396, *P* = 0.002, resp.). Similarly, all of the indices of 24 h SBPV were significantly associated with the IMT (*r* = 0.399, *P* = 0.002; *r* = 0.376, *P* = 0.003; *r* = 0.339, *P* = 0.008, resp.). Conversely, there is no significant correlation between the nighttime BPV and carotid IMT. In addition, for the daytime DBPV and 24 h DBPV, both SD and CV indices were significantly related to IMT (for daytime DBPV, *r* = 0.293, *P* = 0.023; *r* = 0.302, *P* = 0.019, resp.; for 24 h DBPV, *r* = 0.328, *P* = 0.010; *r* = 0.323, *P* = 0.012, resp.), which differed from those evaluated with ARV. To further compare the results, we described these correlations in Figure 1. As Figure 1 showed, the SBPV during daytime and 24 h had greater correlation than DBPV during daytime and 24 h. Moreover, the correlations of the SBPV (evaluated with SD, CV, and AVR) and IMT were almost the same. However, for the DBPV during the daytime and 24 h, the SD and CV indices of BPV had greater correlation with IMT than ARV index.

The correlations between the average BP values and carotid IMT/number of plaques were analyzed using Pearson's test. The results were shown in Table 4. It indicated that there was no significant correlation between these BP variables and carotid IMT, whereas, 24 h PP, daytime PP, and nighttime PP are positively associated with the number of plaques (*r* = 0.349, *P* = 0.006; *r* = 0.332, *P* = 0.01; *r* = 0.370, *P* = 0.004, resp.). Moreover, a negative correlation was found between the nighttime average DBP and the number of plaques (*r* = −0.254, *P* = 0.05).

**Figure 2:**
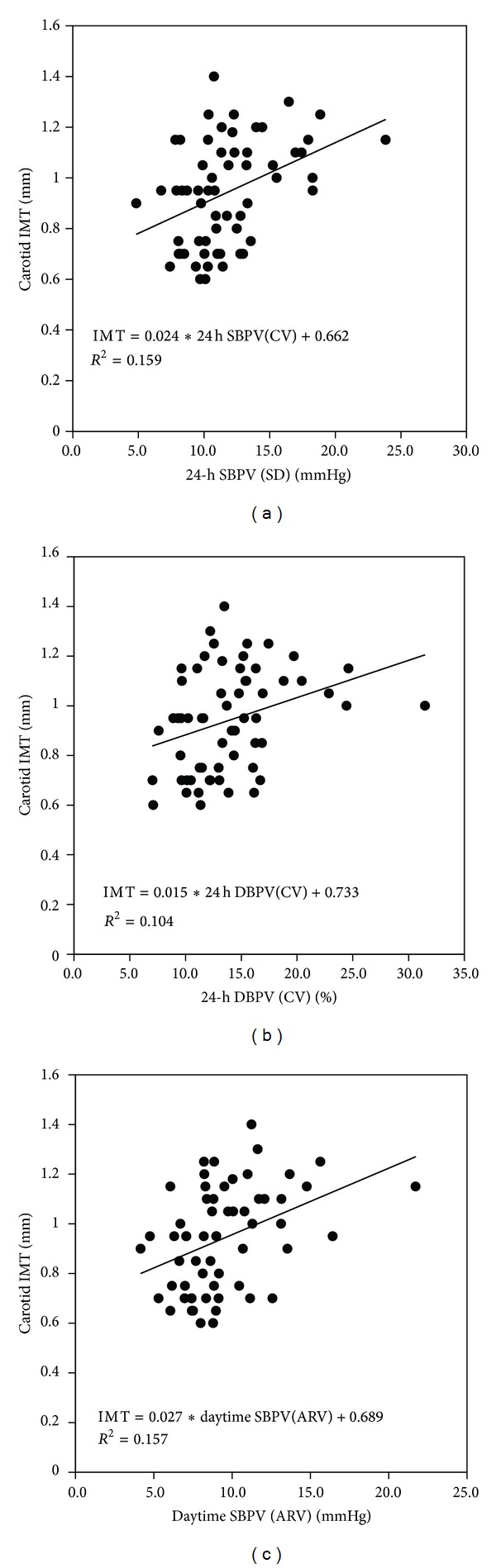
Linear correlation between common carotid artery intima-media thickness (IMT) and 24 h systolic blood pressure variability (SBPV) (SD), 24 h diastolic blood pressure variability (DBPV) (CV) and daytime systolic blood pressure variability (SBPV) (ARV).

Before evaluating the effects of different indices of BPV on the carotid IMT, a simple linear regression analysis was performed for each BPV index. 24 h SBPV (SD), 24 h DBPV (CV), and daytime SBPV (ARV) presented the highly linear correlations with carotid IMT, which were shown in Figure 2.

To further compare the effects of different indices of BPV on IMT, multiple linear regression analyses were performed using the backward selection method for different models in Table 5. *P* value of 0.1 or less was the criterion for a variable to remain in the model, and *P* value less than 0.05 was considered statistically significant. In the multiple regression models (model 1 to model 3), we set carotid IMT as dependent variable and daytime, nighttime, 24 h SBPV, and DBPV evaluated with different BPV indices as independent variables, respectively. The results showed that, for the SD index, 24 h SBPV was the only variable maintained in model 1 (*P* = 0.002, *R* ^2^ = 0.159). For the CV index, 24 h SBPV remained in model 2 (*P* = 0.003; *R* ^2^ = 0.141). For the ARV index, daytime SBPV is the most associated with carotid IMT (*P* = 0.002, *R* ^2^ = 0.157). Afterward, these significant independent factors from model 1 to model 3 entered into the mixed model as the independent factors and carotid IMT also as the dependent variable. We found that daytime SBPV (ARV) was the only significant factor (*P* = 0.002) remaining in the mixed model. In addition, every BPV variable entered into the multiple linear regression models which are after adjusting for the baseline characteristics and corresponding mean BP and PP values (please see Table 6, and the SBPV and DBPV values are displayed in bold fonts). We found that there were no obvious differences among three SBPV variables. However, the SD index of 24 h DBPV revealed better performance unexpectedly.

## 4. Discussion

The previous studies found that the short-term BPV had some effects on the carotid artery alteration (increased IMT and arteriosclerosis) and plaque formation [20–22]. However, the BPV within 24 h can be evaluated by different indices. It is unclear if the effects induced by different indices of BPV can be equal. In our present study, we have found that the SBP fluctuations during daytime and 24 h were significantly associated with the increased carotid IMT. Moreover, from the multiple linear regression models using different indices of BPV as the independent factors, we pointed out that daytime SBPV in ARV index had generally stronger relationship with IMT than SD or CV. It indicated that BPV evaluated with ARV was easier to reflect the effects on the carotid IMT. After adjusting by age, sex, smoking, hypertension, and corresponding mean BP and PP values, we found that the 24 h DBPV evaluated with SD presents the favorable performance in the regression model.

Our first finding corroborates the recently reported results of García-García et al. [16], who examined the relationship of 24 h BPV with vascular structure and function using SD and CV. In their study, both SD and CV of SBP during daytime had significant correlation with IMT (*r* = 0.260, *P* < 0.001; *r* = 0.187, *P* < 0.001); on the contrary, the significant correlations had not been found in DBPV and IMT. The similar conclusions were proposed from the European Lacidipine Study on Atherosclerosis (ELSA) [23]. They indicated that the IMT positively associated with not only 24 h, day, and night average SBP and PP but also the SD of 24 h SBP and PP. However, the relationships between DBPV and IMT were not referred in both of their studies. In our study, the results of correlation analysis were in agreement with their studies, mainly for the relationship between SBPV and IMT. Besides, a new finding in our study is that both daytime DBPV and 24 h DBPV evaluated with SD and CV are associated with IMT. These findings are consistent with some of the conclusions of Shintani et al. [24]. General Japanese population was analyzed in their Ohasama study, and they found that daytime and nighttime DBPV (SD) were both significantly related to the mean IMT (*r* = 0.17, *P* < 0.0001; *r* = 0.15, *P* < 0.0001, resp.). However, only daytime DBPV (*P* = 0.015) maintained the significant correlation with the mean IMT after adjustment for BP level and the 24 h DBPV analysis was not referred.

Besides, one major contribution of our study is to compare the effects on the carotid artery structure for different indices of BPV. Firstly, we found that no significant differences in means ± SDs were documented among three indices of BPV. However, their correlations with IMT were mostly different, which was further demonstrated in the multiple linear regression analysis. Overall, these findings support the view that the prognostic importance of BPV will be affected by the indices of BPV [25]. Nowadays, most of the studies explored the prognostic significance of BPV for the cardiovascular events, but not the carotid artery alteration or arteriosclerosis. In the population-based study in Latin America [26], Mena et al. found that the commonly used SD index may be more sensitive to the sampling frequency of the ABPM devices, and ARV index (*RR* = 1.611, *P* = 0.004) is a more reliable representation of time series variability than SD (*RR* = 1.103, *P* = 0.571) for the prognostic significance of BPV. The similar conclusions have been shown in the study of Stolarz-Skrzypek et al. [27] and Hansen et al. [28]. In our present study, we not only found the most significant BPV variable to reflect the alteration of carotid IMT for each BPV index but also compared the effects of these BPV variables in the mixed model. Only daytime SBPV (ARV) remained after the backward selection regression. Moreover, the association remainned significant even after adjusting for the baseline characteristics and the corresponding mean BP values. Besides, 24 h DBPV and daytime DBPV (SD and CV) were also correlated with the IMT and contributed to the multiple linear regression models after adjustment, which is the new finding of our present study. We speculated the reasons from the calculating formulae of different indices of BPV. SD can only reflect the fluctuation of the BP value. The CV allows the measurement of BPV and eliminates the measurement magnitude effect [16]. However, ARV can reflect the beat-to-beat variation, which is a reliable index for the time series variability [26], especially for the high-sampling BP measurements. However, SD and CV could better present the prognostic significance of DBPV than ARV.

Other findings of our study also deserve to be discussed. Firstly, daytime PP, nighttime PP, and 24 h PP are positively associated with the number of plaques but not with carotid IMT. Moreover, the nighttime average DBP had negative correlation with the number of plaques in the carotid artery. It is consistent with the conclusions of Shintani et al.; nighttime BPV reflects the prevalence of carotid plaque more precisely than daytime BPV in general population [24]. We previously showed that the increased PP is of prognostic importance in the target-organ damage and plaque formation, especially for the cases with the high SBP in the daytime and low DBP in the nighttime. It can be speculated from the physiological mechanism. Oscillatory shear stress had a major effect on regulating the function of human endothelial cell and it stimulates mononuclear leukocyte adhesion and migration in the arterial wall, which is the initiation of the atherosclerotic lesions [29]. Thus, the increased BP variation is assumed to increase oscillatory shear stress in the walls of the carotid artery. Moreover, the blood vessels are damaged by the oscillatory shear stress and it will finally lead to the carotid plaque formation [24].

Secondly, weather the BPV can be treated as one risk factor independent to the average BP level for arteriosclerosis or cardiovascular events are still not clear in previous studies. Mancia et al. have found that the end-organ damage was determined not only by the degree of the high BP level but also by the magnitude of the BPV occurring over the 24 h period [9, 11]. In addition, Kikuya et al. mentioned that BPV was an independent predictor for cardiovascular mortality in the general population [9]. However, Hansen et al. stated that BPV remained an elusive predictor of cardiovascular outcome, as it was strongly associated with the BP level [30]. In a recent published paper, Nagai et al. found that SD, CV, and delta (maximum-minimum) in visit-to-visit SBP were associated with the max-IMT for the high-risk elderly, whereas only delta SBP had significant correlation with max-IMT independently of average SBP [31]. In our multiple linear regression analysis, it has been demonstrated that all of the BPV variables were not independent of the mean BP and PP values for evaluating the increased carotid IMT but independent of the well-known confounding factors, such as age, smoking, and hypertension. Despite all this, more evidence is still required to assess whether the BPV is an independent predictor of arteriosclerosis or cardiovascular events.

Finally, certain limitations of our study should be briefly discussed. First, the relatively small size of the subjects is the main limitation in our present preliminary study. Although the statistically significant results were obtained in our study and the results can be explained by the physiological mechanism, the number of subjects could be still too small to extrapolate the study findings to general population in the southern area of China. The second limitation is the precision of calculating BPV during nighttime. We set the sampling frequency of ABPM according to the BP monitoring protocol constituted by the Cardiac Electrocardiogram Room, the Second Peoples' Hospital of Shenzhen. Although one-hour interval sampling during nighttime may not disturb the sleeping of patients, it is really an exceedingly low sampling frequency. Once one or more measurements are invalid or lost, the calculated results of BPV will be affected to some extent. Lastly, in our present study, the independent factors are limited to the baseline characteristics (age, sex, hypertension, and smoking status), average BP values, and BPV variables. It may lose some significant variables which can contribute to the carotid artery alteration. Meanwhile, the influences of confounding variables deserve to be discussed, although we utilized the backward regression to reduce the influence of the confounding variables.

In conclusion, though the number of samples in this compared study was limited, we also obtained the suggestive conclusions on the relationship between ambulatory BPV and carotid IMT. Our findings extended the validation of the correlation of ambulatory BPV and carotid artery alterations from western population study to the population in the southern area of China.

## 5. Conclusions 

In this study, we provide the evidence that, for the subjects from the southern area of China, all of the indices of SBPV for daytime and 24 h had significant correlation with IMT. Among them, daytime SBPV evaluated with ARV can be the best variable to explain the increase of carotid IMT in linear regression model. In addition, daytime DBPV and 24 h DBPV (SD and CV) are also found to be related to the increased IMT. We consider that ARV is a better representation of BPV than SD and CV for the prognostic significance of carotid artery alteration. However, a longitudinal study is needed to verify the prognostic value of different indices of BPV for the cardiovascular events. In the future, we will conduct the large-scale trials and perform multivariate analysis to investigate how to predict the risks of CVD and mortality from the alteration of carotid structure and function.

## Figures and Tables

**Table 1:** Clinical characteristics of all the subjects and the two subgroups: normal IMT group and abnormal IMT group.

Characteristics	All subjects (*N* = 60)	IMT < 1.0 mm (*N* = 26)	IMT ≥ 1.0 mm (*N* = 34)	*P* value
Age (years)	58.7 ± 12.1	52.4 ± 10.6	63.2 ± 11.1	0.000**
Male gender (%)	53.3	38.5	64.7	0.273
Smoking (%)	23.3	11.5	32.4	0.573
IMT ≥ 1.0 mm (%)	56.7	0	100	<0.001**
Presence of plaque (%)	46.7	19.2	67.6	<0.001**
Hypertensive patients (%)	76.7	69.2	82.4	0.196
CCA IMT (mm)	0.9 ± 0.2	0.76 ± 0.11	1.08 ± 0.15	<0.001**
Bulb IMT (mm)	0.8 ± 0.3	0.62 ± 0.10	0.89 ± 0.29	<0.001**
ICA IMT (mm)	0.6 ± 0.1	0.54 ± 0.08	0.64 ± 0.10	<0.001**
24 h SBP (mmHg)	120.2 ± 16.3	121.3 ± 17.4	119.3 ± 15.6	0.813
24 h DBP (mmHg)	76.6 ± 12.9	78.4 ± 14.4	75.2 ± 11.6	0.148
24 h PP (mmHg)	43.6 ± 9.2	42.8 ± 7.6	44.1 ± 10.4	0.142
Daytime SBP (mmHg)	121.2 ± 16.0	122.1 ± 17.0	120.4 ± 15.4	0.763
Daytime DBP (mmHg)	77.5 ± 12.8	79.2 ± 14.2	76.2 ± 11.7	0.131
Daytime PP (mmHg)	43.7 ± 9.1	43.0 ± 7.8	44.2 ± 10.1	0.125
Nighttime SBP (mmHg)	116.7 ± 18.7	118.6 ± 19.2	115.2 ± 18.4	0.860
Nighttime DBP (mmHg)	73.5 ± 13.6	76.1 ± 15.1	71.5 ± 12.2	0.204
Nighttime PP (mmHg)	43.2 ± 10.4	42.4 ± 8.0	43.8 ± 11.9	0.234
SBP decrease (%)	3.6 ± 6.8	3.5 ± 5.5	3.8 ± 7.8	0.838
DBP decrease (%)	5.0 ± 6.9	4.5 ± 5.0	5.3 ± 8.1	0.723

IMT: intima-media thickness; CCA: common carotid artery; ICA: internal carotid artery; SBP: systolic blood pressure; DBP: diastolic blood pressure; PP: pulse pressure. **Correlation is significant at the 0.01 level.

**Table 2:** The blood pressure variabilities evaluated with SD, CV, and ARV in all of the subjects (*N* = 60).

Variables	SD (mmHg)		CV (%)		ARV (mmHg)	
	Mean ± SD	*r* (*P*)^1^	Mean ± SD	*r* (*P*)^2^	Mean ± SD	*r* (*P*)^3^
Daytime SBPV	11.5 ± 3.7	0.879 (<0.001)	9.6 ± 2.7	0.717 (<0.001)	9.5 ± 3.1	0.819 (<0.001)
Daytime DBPV	9.0 ± 2.3	0.458 (<0.001)	13.6 ± 4.6	0.439 (<0.001)	8.0 ± 2.2	0.660 (<0.001)
Nighttime SBPV	9.8 ± 3.7	0.765 (<0.001)	9.2 ± 3.5	0.643 (<0.001)	10.0 ± 4.1	0.805 (<0.001)
Nighttime DBPV	8.4 ± 2.8	0.526 (<0.001)	13.9 ± 5.4	0.383 (0.002)	9.3 ± 3.1	0.734 (<0.001)
24 h SBPV	11.7 ± 3.5	0.889 (<0.001)	9.9 ± 2.8	0.773 (0.001)	9.5 ± 2.9	0.880 (<0.001)
24 h DBPV	9.2 ± 2.0	0.449 (<0.001)	14.0 ± 4.5	0.390 (0.002)	8.3 ± 2.0	0.675 (<0.001)

SD: standard deviation; CV: coefficient of variation; ARV: average real variability; SBPV: systolic blood pressure variability; DBPV: diastolic blood pressure variability. ^1^The correlation between the BPV evaluated with SD and CV; ^2^the correlation between the BPV evaluated with CV and ARV; ^3^the correlation between the BPV evaluated with SD and ARV.

**Table 3:** The correlation between the blood pressure variability (evaluated with SD, CV, and ARV) and carotid intima-media thickness in all the subjects.

Variables	SD		CV		ARV	
	*r *	*P* value	*r *	*P* value	*r *	*P* value
Daytime SBPV	0.408	0.001**	0.381	0.003**	0.396	0.002**
Daytime DBPV	0.293	0.023*	0.302	0.019*	0.177	0.177
Nighttime SBPV	0.087	0.508	0.192	0.142	0.075	0.567
Nighttime DBPV	0.159	0.226	0.234	0.072	0.123	0.351
24 h SBPV	0.399	0.002**	0.376	0.003**	0.339	0.008**
24 h DBPV	0.328	0.010**	0.323	0.012*	0.214	0.101

Abbreviations as in Table 2. *Correlation is significant at the 0.05 level. **Correlation is significant at the 0.01 level.

**Table 4:** The correlation between mean blood pressure values and carotid intima-media thickness/number of plaques in all the subjects.

Variables	IMT		Number of plaques	
	*r *	*P* value	*r *	*P* value
24 h SBP	0.017	0.895	0.005	0.972
24 h DBP	−0.099	0.451	−0.245	0.059
24 h PP	0.169	0.196	0.349	0.006**
Daytime SBP	0.016	0.904	−0.006	0.964
Daytime DBP	−0.099	0.454	−0.244	0.060
Daytime PP	0.171	0.191	0.332	0.010**
Nighttime SBP	0.006	0.963	0.016	0.901
Nighttime DBP	−0.105	0.423	−0.254	0.050*
Nighttime PP	0.157	0.231	0.370	0.004**
SBP decrease (%)	−0.031	0.816	−0.075	0.570
DBP decrease (%)	−0.007	0.959	0.051	0.700

Note: correlation coefficients (*r*) by Pearson's test are shown.

The abbreviations as in Table 1.

*Correlation is significant at the 0.05 level (2-tailed). **Correlation is significant at the 0.01 level (2-tailed).

**Table 5:** Multiple linear regression analyses performed by backward regression.

Model	Independent factor	*β*	Lower 95% interval	Upper 95% interval	*P* value	*R* ^2^
Model 1^a^ (SD)	24 h SBPV	0.024	0.009	0.038	0.002	0.159
	Constant	0.662	0.486	0.838	0.000	

Model 2^b^ (CV)	24 h SBPV	0.028	0.010	0.045	0.003	0.141
	Constant	0.671	0.488	0.853	0.000	

Model 3^c^ (ARV)	Daytime SBPV	0.027	0.010	0.043	0.002	0.157
	Constant	0.689	0.527	0.851	0.000	

Model 4^d^ (Mix)	Daytime SBPV (ARV)	0.027	0.010	0.043	0.002	0.157
	Constant	0.689	0.527	0.851	0.000	

^a^Model 1: using the SDs of blood pressure as the independent factors. ^b^Model 2: using the coefficients of variation of blood pressure as the independent factors. ^c^Model 3: using average real variabilities of blood pressure as the independent factors. ^d^Model 4: backward regression using the significant independent factors from model 1 to model 3.

**Table 6:** Adjusted multiple regression analysis of carotid intima-media thickness and blood pressure variabilities*.

Factors	*β* (95% CI)	*P* value	*R* ^2^
Age^+^	0.069 (0.033–0.105)	0.061	0.449
Smoking	0.150 (0.049–0.251)	0.004	
Hypertension	0.148 (0.047–0.249)	0.005	
24 h SBPV (SD)	**0.016 (0.004**–**0.029)**	**0.012**	

Age^+^	0.066 (0.029–0.103)	0.001	0.402
Smoking	0.150 (0.048–0.251)	0.005	
Hypertension	0.166 (0.064–0.268)	0.011	
24 h SBPV (CV)	**0.019 (0.004**–**0.035)**	**0.017**	

Age^+^	0.073 (0.036–0.109)	0.000	0.424
Smoking	0.136 (0.032–0.240)	0.012	
Hypertension	0.133 (0.027–0.238)	0.015	
Daytime SBPV (ARV)	**0.015 (0.000**–**0.029)**	**0.050**	

Age^+^	0.081 (0.047–0.116)	0.000	0.440
Smoking	0.124 (0.020–0.228)	0.020	
Hypertension	0.136 (0.033–0.239)	0.011	
24 h DBPV (SD)	**0.026 (0.004**–**0.048)**	**0.020**	

Age^+^	0.071 (0.034–0.107)	0.000	0.427
Smoking	0.150 (0.047–0.253)	0.005	
Hypertension	0.163 (0.060–0.267)	0.003	
24 h DBPV (CV)	**0.010 (0.000**–**0.020)**	**0.042**	

CI: confidence interval. *Adjusted by age, sex, smoking, hypertension, and mean BP values. ^+^Per 10-year increase.
